# Using a state cancer registry to recruit young breast cancer survivors and high-risk relatives: protocol of a randomized trial testing the efficacy of a targeted versus a tailored intervention to increase breast cancer screening

**DOI:** 10.1186/1471-2407-13-97

**Published:** 2013-03-01

**Authors:** Maria C Katapodi, Laurel L Northouse, Ann M Schafenacker, Debra Duquette, Sonia A Duffy, David L Ronis, Beth Anderson, Nancy K Janz, Jennifer McLosky, Kara J Milliron, Sofia D Merajver, Linh M Duong, Glenn Copeland

**Affiliations:** 1 University of Michigan School of Nursing, 400 N. Ingalls Building, Room 2158, Ann Arbor, MI, 48109, USA; 2 Michigan Department of Community Health, Cancer Genomics Program, Lansing, USA; 3 University of Michigan School of Nursing and VA Hospital, Ann Arbor, USA; 4 University of Michigan School of Nursing and VA Center for Clinical Management Research, Ann Arbor, USA; 5 University of Michigan School of Public Health, Ann Arbor, USA; 6 University of Michigan Comprehensive Cancer Center, Ann Arbor, USA; 7 University of Michigan School of Medicine and Comprehensive Cancer Center, Ann Arbor, USA; 8 Cancer Surveillance Branch, Division of Cancer Prevention and Control, Centers for Disease Control and Prevention, Atlanta, USA; 9 Michigan Cancer Surveillance Program, Lansing, USA

**Keywords:** Breast cancer screening, Familial breast cancer, Young breast cancer survivors, High-risk relatives, Randomized trial, Targeted and enhanced tailored intervention, Screening mammography, Genetic testing, Cancer registry, State-wide community-based sample

## Abstract

**Background:**

The Michigan Prevention Research Center, the University of Michigan Schools of Nursing, Public Health, and Medicine, and the Michigan Department of Community Health propose a multidisciplinary academic-clinical practice three-year project to increase breast cancer screening among young breast cancer survivors and their cancer-free female relatives at greatest risk for breast cancer.

**Methods/design:**

The study has three specific aims: 1) Identify and survey 3,000 young breast cancer survivors (diagnosed at 20–45 years old) regarding their breast cancer screening utilization. 2) Identify and survey survivors’ high-risk relatives regarding their breast cancer screening utilization. 3) Test two versions (Targeted vs. Enhanced Tailored) of an intervention to increase breast cancer screening among survivors and relatives. Following approval by human subjects review boards, 3,000 young breast cancer survivors will be identified through the Michigan Cancer Registry and mailed an invitation letter and a baseline survey. The baseline survey will obtain information on the survivors’: a) current breast cancer screening status and use of genetic counseling; b) perceived barriers and facilitators to screening; c) family health history. Based on the family history information provided by survivors, we will identify up to two high-risk relatives per survivor. Young breast cancer survivors will be mailed consent forms and baseline surveys to distribute to their selected high-risk relatives. Relatives’ baseline survey will obtain information on their: a) current breast cancer screening status and use of genetic counseling; and b) perceived barriers and facilitators to screening. Young breast cancer survivors and high-risk relatives will be randomized as a family unit to receive two versions of an intervention aiming to increase breast cancer screening and use of cancer genetic services. A follow-up survey will be mailed 9 months after the intervention to survivors and high-risk relatives to evaluate the efficacy of each intervention version on: a) use of breast cancer screening and genetic counseling; b) perceived barriers and facilitators to screening; c) self-efficacy in utilizing cancer genetic and screening services; d) family support related to screening; e) knowledge of breast cancer genetics; and f) satisfaction with the intervention.

**Discussion:**

The study will enhance efforts of the state of Michigan surrounding cancer prevention, control, and public health genomics.

**Trial registration:**

NCT01612338

## Background

Breast cancer is the second most common cancer among U.S. women and the second leading cause of cancer death [1]. One of the goals of the Comprehensive Cancer Control Plan for Michigan is to further “reduce the female breast cancer death rate” [2]. This study strives to contribute to this goal, specifically for female Young Breast Cancer Survivors (YBCS) diagnosed at 20–45 years old and their female relatives who may be at increased risk.

Breast cancer survivors have a 2-fold higher risk of developing a second breast cancer, compared to women without breast cancer, matched for age, breast density, and use of mammography [3]. In addition, unaffected first- and second-degree relatives of women diagnosed with breast cancer younger than 50 years of age have respectively a 2.3 and 1.5 increased relative risk for breast cancer [4]. The initial step in determining an unaffected woman’s risk for breast cancer is the collection of a thorough family history that includes first- and second-degree relatives on both the maternal and paternal sides of the family [5]. However, a review of health plan charts conducted by the Genomics Program at the Michigan Department of Community Health revealed that only 42% of charts had documented a family history of breast cancer, while, 98% of these charts did not document age of onset for the affected family members [6]. According to national recommendations, women with a strong family history of breast cancer should be referred for genetic counseling [7,8]. Yet, a phone survey of the general adult population living in Michigan revealed that only 12% of high-risk women older than 40 years of age actually received this service [6]. Furthermore, only 56% of these high-risk women had a Clinical Breast Exam (CBE) in the past 12 months; only 48% had a mammogram in the past 12 months; and, only 44% had both a mammogram and CBE in the past 12 months [6].

The study aims to increase breast cancer surveillance and early detection by targeting YBCS identified from a state cancer registry and their high risk relatives. Specific aims are to:

Aim 1: Identify and survey 3,000 female YBCS reported to the cancer registry who were diagnosed between the ages of 20–45 years and determine: (a) their current breast cancer screening status; (b) perceived barriers and facilitators to screening; (c) willingness to participate in an intervention to increase breast cancer screening; and (d) willingness to serve as a breast cancer screening advocate for their high-risk relatives.

Aim 2: Identify and survey up to two unaffected first- and/or second-degree female relatives per YBCS and determine: (a) their current breast cancer screening status; (b) perceived barriers and facilitators to screening; and (c) willingness to participate in an intervention to increase breast cancer screening. Female relatives will be between 25–64 years and have an increased risk of breast cancer based on the YBCS’ age of diagnosis.

Aim 3: Compare the efficacy of two versions of an intervention on breast cancer screening utilization and other outcomes among YBCS and their high-risk female relatives. YBCS and their high-risk female relatives will be randomly assigned as a family unit to receive either the Targeted or the Enhanced Tailored version of the intervention. (Description of the two intervention methods follows).

## Methods/design

This prospective, randomized trial involves testing the efficacy of two versions of a printed intervention (i.e., Targeted version and Enhanced Tailored version). Participants will be randomly assigned as family units (YBCS and her high-risk female relatives) to receive one of two versions. The survivor and relative(s) of enrolled family units will be mailed a self-administered baseline survey prior to receiving the intervention (Time 1). A self-administered, follow-up survey will be mailed to YBCS and high-risk relatives nine months post-intervention (Time 2).

### Setting

The Michigan Cancer Surveillance Program is a central cancer registry that was established by law (Act 82 of 1984) [9] to collect reports on cases for *in situ* and invasive malignancies. Between 1998 and 2007, there were 7,866 YBCSs identified in the Michigan Cancer Surveillance Program [10]. This database will be used to identify and recruit YBCS. The Michigan Department of Community Health-Genomics Program and the University of Michigan-School of Nursing will enroll participating families, implement the two versions of the intervention, and collect and analyze baseline (Time 1) and follow up (Time 2) data.

### Recruitment of YBCS and high-risk relatives

**Figure 1:**
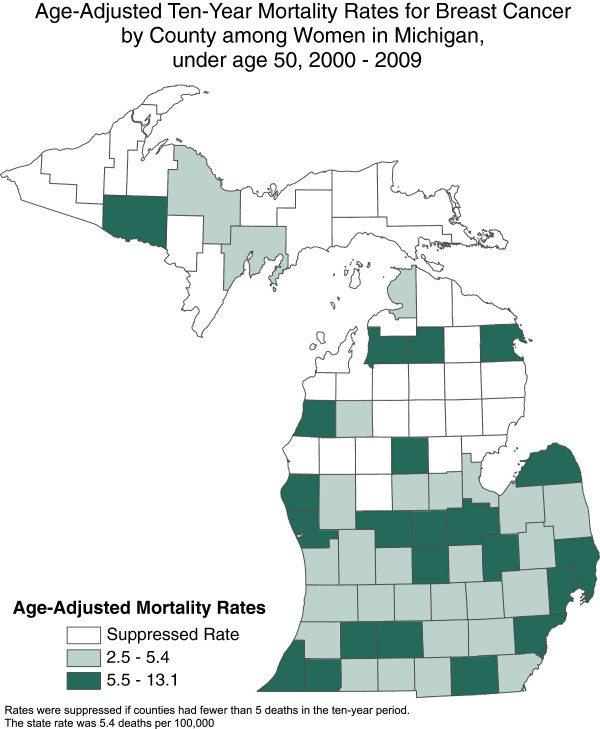
Age-adjusted ten-year mortality rates for breast cancer by county among women in Michigan under age 50, 2000–2009.

Between 1994 and 2008 there were 9,000 cases of young women with breast cancer were reported to the Michigan Cancer Registry. For the study, 3,000 women diagnosed with invasive or in –situ-breast cancer between 20 to 45 years old from 1994 to 2008 will be randomly selected from the cancer registry database. The Michigan Cancer Surveillance Program will cross-reference mortality data to exclude deceased YBCS. To increase inclusion of minority and underserved women, the sample will be stratified by race. The study will oversample YBCS who are black and living in counties with the highest mortality rates for young women with breast cancer (See Figure 1).

**Figure 2:**
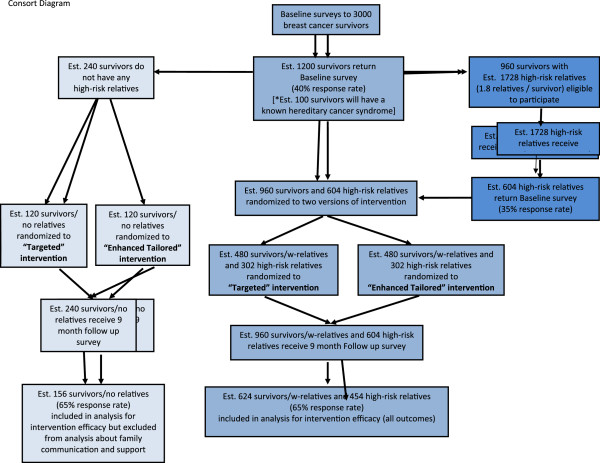
**CONSORT diagram-flow of study participants.** Est. = Estimated.

As shown in Figure 2, it is estimated that from the initial 3,000 YBCS, approximately 1,200 will be willing to participate (40% response rate). We estimate that approximately 20% of these YBCS (n = 240) will not have any eligible high-risk female relatives (e.g. relatives will be younger than 25 or older than 64). These YBCS will be included in the study but will be analyzed as a separate group. Based on our previous experience recruiting women with familial breast cancer and their high-risk relatives [11], we project that the remaining 960 YBCS will have approximately 1,728 eligible high-risk female relatives. This estimation is based on the assumption that we will be able to identify 1.8 first- and/or second-degree, high-risk relatives per YBCS. Based on our previous experience [11], we expect that 604 high-risk relatives will be willing to participate in the study (35% response rate). Table 1 describes inclusion and exclusion criteria for YBCS and high-risk female relatives.

**Table 1:** Eligibility criteria

**Eligibility criteria for young breast cancer survivors**	**Eligibility criteria for high-risk female relatives**
• Female	• Female
• 25–64 years of age	• 25–64 years of age
• Being diagnosed with unilateral or bilateral invasive breast cancer between 20 and 45 years old	• Unaffected with any type of cancer
• Being diagnosed with unilateral or bilateral DCIS^ж^ between 20 and 45 years old	• First- or second-degree relatives of the YBCS
• Michigan resident at time of diagnosis	• US resident
• Able to read and understand English	• Able to read and understand English
• Not currently pregnant, incarcerated, or institutionalized*	• Not currently pregnant, incarcerated, or institutionalized*
	• YBCS is willing to contact

^ж^ Ductal Carcinoma In Situ.

* Women who are pregnant, incarcerated, or institutionalized at the time of the study are excluded because they may not be able to follow recommendations for breast cancer screening and genetic counseling.

Recruitment procedures will involve the following steps:

1) Obtain approval to conduct research with human subjects from the Institutional Review Boards at the University of Michigan and at the Michigan Department of Community Health, and the Scientific Advisory Board of the Michigan Cancer Registry.

2) The cancer registry will send a letter to the reporting facility and the physician of record asking if they are aware of any reason that the YBCS should not be contacted to participate in the study. The YBCS will be excluded from the study if the reporting facility or physician of record responds to this letter requesting that the YBCS not be contacted.

3) If there is no reason to exclude YBCS, the cancer registry will mail an invitation letter to the YBCS requesting her participation in the study, along with an Informed Consent form, and the self-administered baseline survey. The invitation letter and consent form will explain the study and state that if the YBCS is currently incarcerated or institutionalized, or if she is pregnant, she is not eligible to participate. The invitation letter will explain that these two conditions may interfere with a woman being able to get breast cancer screening. The invitation letter and consent form include contact information for the Director of the cancer registry and the Principal Investigator of the study, and a toll-free phone number for the Michigan Department of Community Health-Genomics Program for the YBCS to ask further questions about the study.

4) YBCS who agree to participate will return the signed consent form and the completed baseline survey to the cancer registry in a postage-paid, pre-addressed envelope. There will be up to three mailed attempts to reach non-responding YBCS. The cancer registry will not release any identifiable information to the research team until the YBCS mails back her signed consent form and her baseline survey.

5) Once an YBCS agrees to participate, her contact information and baseline survey will be released to the Michigan Department of Community Health-Genomics Program. Two board-certified genetic counselors employed by the program will review all returned surveys to ascertain YBCS eligibility. If the YBCS reports being diagnosed with a known hereditary cancer syndrome such as *BRCA1*, *BRCA2*, Lynch, *PTEN* Hamartoma Tumor, Li-Fraumeni, or Peutz-Jeghers syndromes, the genetic counselors will contact her by phone and provide additional information to raise awareness about appropriate resources and clinical care. These YBCS (estimated n = 100) will be excluded from Aim 3 of the study.

6) Based on information provided by the YBCS in the baseline survey, the genetic counselors will identify up to two high-risk relatives per YBCS. Eligible high-risk relatives will be female, first- and/or second-degree relatives, and unaffected by cancer. Identification of high-risk relatives will be according to a protocol that involves pedigree analysis. Questions that assess family history of cancer allow calculations of Gail [12] and Claus [13] risk models. YBCS will be asked how many first and second degree relatives had cancer, type of cancer, and age of onset. Then YBCS are asked to list the first and second degree relatives in the family who are cancer free, their age, and whether they are willing to contact them for the study. The combination of answers in these two sets of questions allows genetic counselors to identify eligible, high-risk relatives. As explained in the informed consent form, if necessary, the genetic counselors will contact the YBCS by phone to obtain additional family history information. Each participating YBCS will be mailed a letter asking her to contact the identified high-risk relatives and request their participation in the study. Consent forms, baseline surveys and postage-paid return envelopes will be provided to the YBCS to give to her relatives. A “Project Navigator” will be available by telephone to discuss any concerns the YBCS may have about this procedure. If an identified high-risk relative does not mail back her signed consent form and completed baseline survey within six to eight weeks, the Project Navigator will contact the YBCS to determine if an alternate high-risk relative should be selected for participation.

7) Relatives’ signed consents and completed baseline surveys will be returned to the Michigan Department of Community Health-Genomics Program. This is the first time that the research team will receive identifiable information from high-risk relatives. The genetic counselors will review relatives’ signed consent forms to further ascertain eligibility for participation. After randomization, the genetic counselors will also calculate objective breast cancer risk for the high-risk relatives who will be randomized to receive the Enhanced Tailored version of the intervention. The Gail model [12] and the Claus model [13] will be used to calculate these objective risk estimates. Using two different risk estimation models is necessary because the Gail model does not apply to women younger than 35 years of age.

8) YBCS and high-risk relatives will receive a check for $10 in the mail when they return their baseline survey and a check for $20 when they return their follow-up survey.

### Stratification and randomization

Before randomization, YBCS (with and without high-risk relatives) will be stratified by self-reported race (black vs. other) to ensure equal distribution of ethnic minority participants across study arms. YBCS (n = 960 with high-risk relatives and n = 240 without high-risk relatives) will be randomly allocated to receive either the Targeted or the Enhanced Tailored version of the intervention via a computerized program generated by the study statistician. YBCS and high-risk relatives will be randomly assigned as a family unit.

### Targeted vs. enhanced tailored intervention

**Figure 3:**
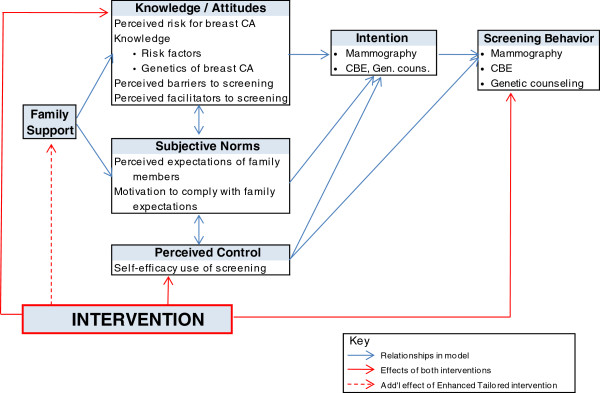
**Expanded theory of planned behavior.** CBE = Clinical Breast Exam.

An expansion of the Theory of Planned Behavior (TPB) [14] was used as the framework to guide the development of the two versions of the intervention. The expanded TPB includes two additional components that are specific to the needs of YBCS and their high-risk relatives. The first component is knowledge about breast cancer risk factors and cancer genetics. The second component is perceived family support regarding breast cancer screening behaviors. Figure 3 shows the modified version of the TPB and the constructs hypothesized to be affected by the two versions of the intervention.

**Figure 4:**
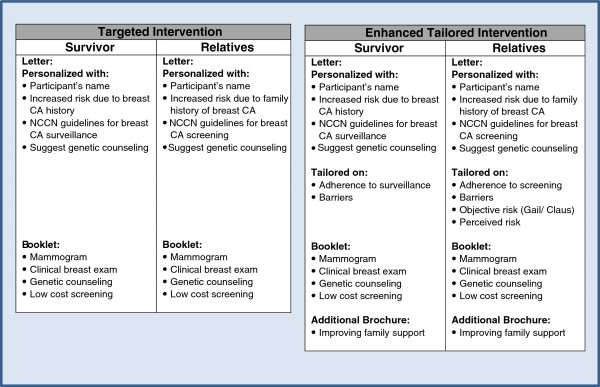
**Components of the targeted and the enhanced tailored version of the intervention.** CA = Cancer.

Development of the Targeted version is based on a mailed intervention recommended by the Guide to Community Preventive Services as efficacious in increasing breast cancer screening among older, non-adherent women [15]. Participants randomized to the Targeted version will receive a personalized letter and a booklet that addresses breast cancer screening and genetic counseling in YBCS and high-risk relatives. The Enhanced Tailored version of the intervention will provide tailored information about 1) breast cancer risk; 2) adherence to screening and perceived barriers; and 3) ways to enhance family support related to breast cancer screening. Based on participants’ responses to the baseline survey, the study team will add evidence-based components to address the specific needs of YBCS and their high-risk female relatives that will be randomized to receive the Enhanced Tailored version. All the components that comprise the two versions of the mailed intervention are shown in Figure 4. All intervention materials are developed at the 9^th^ grade reading level or less.

Tailored health messages have the greatest advantage over targeted messages when there is significant variability within the targeted audience on key determinants of the intended outcome, e.g., knowledge and attitudes [16]. When there is little variability on the key determinants of the intended outcome, targeting could be as effective as tailoring and more cost-effective. In the case of screening mammography, two meta-analyses reported that although tailored interventions are more efficacious compared to non-tailored ones in increasing mammography screening [17,18], reminder-type, targeted interventions could also be efficacious in promoting repeated use of mammography [19]. Given that tailored interventions require a pre-existing mechanism for gathering data from the target population [16] they demand more resources making their routine implementation less likely. The present study will provide information on the level of variability among hypothesized key determinants of breast cancer screening, namely, access barriers and lack of social/family support among YBCS and their high-risk relatives, and it will allow the comparison of the efficacy of each intervention version.

### Outcomes

A follow-up survey will be mailed to YBCS and their high-risk relatives nine months after the baseline survey to determine the effect of each intervention version on: a) utilization of breast cancer screening and genetic counseling; b) perceived barriers and facilitators to screening; c) self-efficacy in utilizing screening services; d) family support related to screening; e) knowledge of the genetics of breast cancer; and f) satisfaction with the intervention. Table 2 describes the concepts and variables of the study (left column), the instruments that will be used to measure these variables (middle column), and the assessment times (right column). Instruments have been previously validated with various populations (see references in Table 2). Completion of the baseline and the follow-up questionnaires takes approximately 45 min.

**Table 2:** Variables, instruments and assessment times

**Concepts/variables**	**Instruments**	**Survivor**		**High-risk relative**	
		**Baseline**	**Follow up**	**Baseline**	**Follow up**
**Knowledge/Attitude**					
Perceived breast cancer risk	Perceived breast cancer risk [11,20]			√	√
Knowledge of breast cancer risk factors	Breast cancer risk factor knowledge index [11,21]	√	√	√	√
Knowledge of breast cancer genetics	Knowledge of breast cancer gene inheritance [22]	√	√	√	√
Perceived facilitators vs. barriers of mammography screening	Decisional balance scale for mammography [23,24]	√	√	√	√
**Subjective norms**					
Perceived family expectations about breast cancer screening	Adapted from Ajzen et al. [14]	√	√	√	√
Motivation to comply with family members’ expectations	Adapted from Ajzen et al. [14]	√	√	√	√
**Family support**					
Perceived family support for breast cancer screening	Social support for breast cancer screening [25]	√	√	√	√
**PERCEIVED CONTROL**					
Self-efficacy in utilizing breast cancer screening services	Self-efficacy for mammography, CBE, cancer genetic services [14]	√	√	√	√
**Intention**					
Intention to pursue mammogram, CBE, genetic counseling (when applicable)	Intention to pursue mammography, CBE, and cancer genetic services (when applicable) [14]	√	√	√	√
**Behavior**					
Current screening practices	Behavioral risk factors surveillance system [24]	√	√	√	√
**Other**					
Demographics/personal and family history	Behavioral risk factors surveillance system [26]	√		√	
Breast cancer risk (Gail model)	Breast cancer risk assessment tool [27]			√	√
Breast cancer risk (Claus model)	Claus breast cancer risk tables [13]				
Evaluation of the Acceptability of the Intervention	TBD by researchers		√		√

### Sample size (power analysis)

Data analyses to meet Aim 3 will require comparison of randomly assigned subsamples of the entire sample and make the most demand on the sample size. PASS software [28] was used to determine the number of participants needed to provide 80% power to detect a medium-small difference (d = 0.3) between means or between percentages (h = 0.3) using a two-tailed test with alpha of 0.05. A medium-small effect size (d = 0.3 or h = 0.3) is a typical effect size found in interventions aiming to increase screening mammography [17,18]. The result of power analysis was 176 participants per group or 352 in total. Assuming 35% attrition between baseline and follow up, 542 participants must be given the intervention to maintain this level of power. Given the initial target number for recruitment and the expected response and attrition rates, the study will recruit and maintain a large enough sample size to perform data analyses for Aim 3 (see Figure 3).

### Statistical analyses

Analyses will be conducted separately for YBCS and high-risk relatives. A descriptive analysis of baseline data will provide screening utilization practices, perceived barriers and facilitators to screening, and other outcomes for YBCS and high-risk relatives. This will include tabulating counts and frequencies of variables including demographics, cancer history, screening history and perceived breast cancer risk. Bivariate analyses (using the Chi-square test for differences in proportions and *T*-test for differences in means) to assess the associations between demographic factors, clinical indications and screening practices will follow. We will stratify by using the Cochran-Mantel-Haenszel test to assess the association between family history, screening practices, genetic counseling/testing, and perceived facilitators/barriers while controlling separately for race/ethnicity, age-group, and time since diagnosis. Outcomes include both continuous and dichotomous measures. Means and standard deviations will be used to describe the results. Continuous measures taken only at post-test will be compared between the two intervention groups by simple t-tests. After assuming an autocorrelation of data r = .30, changes over time in continuous measures will be tested by paired t-tests. For dichotomous measures taken only at post-test, the rates in the two groups will be compared by logistic regression. The proportions in each group will be reported to describe the results. If a baseline version of the measure is available, logistic regression will be conducted on the post-tests with the pre-tests included as covariates. Chi-square tests will be conducted to test the changes over time. The above analyses will establish whether the groups are equivalent, determine whether post-tests differ between the two groups, and assess the significance of changes over time. To further meet Aim 3, regression analyses (linear or logistic) will be used to test which factors (beyond the intervention) predict obtaining breast cancer screening.

## Discussion

First, this study will expand public health knowledge about breast cancer surveillance practices among YBCS, their perceived facilitators and barriers to screening, and their willingness to advocate for their high-risk female relatives. Special attention will be given to minority YBCS. The study will allow us to further understand the needs of these high-risk women including barriers to breast cancer screening. Due to random sample selection and random allocation, study findings can be generalized to all YBCS and high-risk relatives in the state of Michigan and possibly to other U.S. states with similar demographic composition and similar availability and accessibility of breast cancer screening services. Second, confirmation of family history will occur simultaneously with identification of the YBCS in the cancer registry followed by outreach to her high-risk female relatives. By circumventing the typical barriers associated with family history collection (i.e., client awareness of family history, provider practices regarding family history collection and referral), the study aims to increase breast cancer screening among women at greatest risk for breast cancer. This innovative approach of identifying high-risk women through existing public health data may lead to a new method of family history collection and breast cancer risk assessment. Third, this study is among the first to evaluate two versions (Targeted vs. Enhanced Tailored) of a printed intervention as a means of increasing breast cancer screening in YBCS and their high-risk female relatives. This novel approach will likely expand our knowledge about interventions that can increase breast cancer screening among women at substantially higher risk.

## Abbreviations

YBCS: Young breast cancer survivor; DCIS: Ductal carcinoma in situ; CA: Cancer.

## Competing interests

The authors declare that they have no competing interests.

## Authors’ contributions

MK developed the two versions of the intervention and the study protocol and will oversee the overall scientific integrity of the study. LN developed the two versions of the intervention and the study protocol. AS developed the two versions of the intervention and the study protocol. SD developed the two versions of the intervention and the study protocol. DD assisted in the development of the study protocol and the intervention and will evaluate family history, identify high-risk relatives, calculate objective breast cancer risk, and act as a “Project Navigator.” DR provided the proposed statistical analyses. BA assisted in the development of the study protocol, methods for subject recruitment, and statistical analyses. NJ provided consultation on the development of the two versions of the intervention. JM assisted in subject eligibility criteria and will evaluate family history, identify high-risk relatives, calculate objective breast cancer risk, and act as a “Project Navigator.” KM assisted in subject eligibility criteria and will evaluate family history, identify high-risk relatives, calculate objective breast cancer risk, and act as a “Project Navigator.” SM provided consultation on subject eligibility criteria and the development of the intervention and the study protocol. LMD reviewed the manuscript and provided comments as a scientific collaborator at the Centers of Disease Control and Prevention. She is collaborating with the Principal Investigator on programmatic issues. GC assisted with methods for study recruitment. All authors contributed to the final manuscript. All authors read and approved the final manuscript.

## Pre-publication history

The pre-publication history for this paper can be accessed here:

http://www.biomedcentral.com/1471-2407/13/97/prepub
